# Hypoxia Induces Autophagic Cell Death through Hypoxia-Inducible Factor 1α in Microglia

**DOI:** 10.1371/journal.pone.0096509

**Published:** 2014-05-12

**Authors:** Zhao Yang, Tian-zhi Zhao, Yong-jie Zou, John H. Zhang, Hua Feng

**Affiliations:** 1 Department of Neurosurgery, Southwest Hospital, Third Military Medical University, Chongqing, China; 2 Department of Neurosurgery, Tangdu Hospital, Fourth Military Medical University, Xi'an, Shaanxi Province, China; University of Dundee, United Kingdom

## Abstract

As phagocytic cells of central nervous system, excessive activation or cell death of microglia is involved in a lot of nervous system injury and degenerative disease, such as stroke, epilepsy, Parkinson's disease, Alzheimer's disease. Accumulating evidence indicates that hypoxia upregulates HIF-1α expression leading to cell death of microglia. However, the exact mechanism of cell death induced by hypoxia in microglia is not clear. In the current study, we showed that hypoxia induced cell death and autophagy in microglia. The suppression of autophagy using either pharmacologic inhibitors (3-methyladenine, bafilomycin A1) or RNA interference in essential autophagy genes (BECN1 and ATG5) decreased the cell death induced by hypoxia in microglia cells. Moreover, the suppression of HIF-1α using either pharmacologic inhibitors (3-MA, Baf A1) or RNA interference decreased the microglia death and autophagy in vitro. Taken together, these data indicate that hypoxia contributes to autophagic cell death of microglia through HIF-1α, and provide novel therapeutic interventions for cerebral hypoxic diseases associated with microglia activation.

## Introduction

Ischemic stroke, the most common acute cerebrovascular disease with high morbidity and mortality, is one of the leading causes of human deaths[1], [2], [3]. The pathogenesis of this disease has not been elucidated yet. Ischemic/hypoxic injury of brain tissues and subsequent necrosis and inflammation of nerve cells had long been considered the principal pathophysiological mechanism of cerebral infarction[4]. Inflammation protects the brain from infection, but it aggravates injury. Furthermore, death of activated microglia (major inflammatory cells in the brain) may regulate brain inflammation [5], [6]. However, the exact mechanism involved in the death of activated microglia under hypoxic is still complex.

Hypoxia-inducible factor 1 (HIF-1) is a key regulator in hypoxia [7], [8], and also is an important player in neurological outcomes following ischemic stroke due to the functions of its downstream genes [9]. These include genes that promote glucose metabolism, angiogenesis, erythropoiesis, and cell survival [10], [11]. During cerebral ischemia, hypoxia may not only directly damage neurons, but also promote neuronal injury indirectly via microglia activation by regulation of HIF-1α [12], [13].

Autophagy, a catabolic digestion process of cellular macromolecules or even whole organelles, plays an important role in protecting cells against adverse conditions such as hypoxia [14], [15]. Autophagy influences the physiological and pathological conditions of many immune cells including macrophages [16]. Autophagy also plays a critical role in the pathogen elimination and cytokines production of macrophages [17]. Therefore, it might be assumed that autophagy pathway plays a role in microglia, the resident immune cells carring many macrophage-like properties in the brain [18]. However, autophagy and its regulation in microglia, and its effect on the production of proinflammatory and cytotoxic factors under hypoxia are largely unknown to date.

In the study, we proposed a hypothesis that autophagy might contribute to cell death of microglia through HIF-1α under hypoxia.

## Materials and Methods

### Antibodies and Reagents

The GFP-MAP1LC3B plasmid was kindly provided by Dr. Tamotsu Yoshimori (Department of Cell Biology, National Institute for Basic Biology, Presto, Japan). 3-methyladenine (3-MA, M9281), Bafilomycin A1 (Baf A1, B1793), 3-(5'-hydroxymethyl-2'-furyl)-1-benzylindazole (YC-1, Y102) and 2-Methoxyestradiol (2ME2, M6383) were purchased from Sigma; antibodies against MAP1LC3B (L7543) and HIF-1α (SAB5200017) was obtained from Sigma. Antibody against BECN1 (612112) was obtained from BD Transduction Laboratories, Inc (Beverly, MA) whereas antibodies against Actin (sc-10731) were obtained from Santa Cruz Biotechnology.

### Microglia cell culture and hypoxia treatment of microglial cells

Cerebral hemispheres of 1-day old postnatal mice were digested with 0.1% trypsin. The cells were seeded into a six-well plate coated with poly-L-lysine and fed with Dulbecco's Modified Eagle Media (DMEM; Sigma, St. Louis, MO, USA) containing 10% fetal bovine serum (FBS; Hyclone, Logan, UT/USA). Culture media were refreshed twice per week for 2 weeks. Microglia were detached by gentle shaking and filtered through a nylon mesh to remove astrocytes. After centrifugation at 1000×g for 10 min, the cells were resuspended in fresh DMEM supplemented with 10% FBS and plated at a final density of 5×10^5^ cells/mL on a poly-L-lysinecoated 6-well culture plate. The following day, cells were subjected to the experiments. The cell purity was determined by immunohistochemical staining using microglia specific antibody CD11b. The microglia cultures used were >95% pure.

For hypoxia treatment, the culture medium was changed to fresh medium for routine culture before the cells were exposed to hypoxia by placing them in a chamber filled with a gas mixture of 2%O2/5% CO2/93% N2 for 2, 4, 16, 24 and 48 hours [19].

### siRNA assay

The HIF-1α (mouse, sc-35562), ATG5 (mouse, sc-41446) and BECN1 siRNAs (mouse, sc-29798) were purchased from Santa Cruz Biotechnology along with control siRNA (sc-44230). All siRNA transfections were performed with Dharmafect 1 transfection reagent (Thermo Scientific, T-2001–03). Microglia was transfected with 50 nM siRNA for 24 h, followed by treatments; protein knockdown were assessed by western blot analysis.

### Transmission Electron Microscopy

Microglia were collected and fixed in a solution containing 2.5% glutaraldehyde in 0.1 M sodium cacodylate for 2 hrs, postfixed with 1% OsO4 for 1.5 hrs, washed and stained in 3% aqueous uranyl acetate for 1 h. The samples were then washed again, dehydrated with a graded alcohol series, and embedded in Epon-Araldite resin (Canemco, #034). Ultrathin sections were cut on a Reichert ultramicrotome, counterstained with 0.3% lead citrate and examined on a Philips EM420 electron microscope.

### Confocal microscopy

Microglia was transfected with the GFP-MAP1LC3B-expressing plasmid. After 24 hrs, cells were cultured with hypoxia for 6 hrs. Cells were washed with PBS and fixed by incubation in 4% paraformaldehyde for 10 min at 37°C. We used a Radiance 2000 laser scanning confocal microscope for confocal microscopy, followed by image analysis with LaserSharp 2000 software (Bio-Rad). Images were acquired in sequential scanning mode.

### Western blot

Cells were washed with ice-cold PBS and then lysed in Triton X-100/glycerol buffer (50 mM TRIS-HCl, 4 mM EDTA, 2 mM EGTA, 1 mM dithiothreitol, and 25% wt/vol sucrose, pH 8.0, supplemented with 1% Triton X-100 and protease inhibitor). After centrifugation at 5,000 g for 15 min at 4°C, the protein concentration was measured with a BCA protein assay kit (Pierce, 23227). Lysates were separated using SDS-PAGE and transferred to polyvinylidene difluoride mem-branes. The membranes were blocked with 5% nonfat dry milk in Tris-buffered saline, pH 7.4, containing 0.05% Tween 20 (Sigma, P1379), and were incubated with primary anti-mouse antibodies and horseradish peroxidase-conjugated secondary anti-mouse antibodies (Jackson Immunoresearch Laboratories, 115-035-003) or anti-rabbit antibodies (Jackson Immunoresearch Laboratories, 111-035-003) according to the manufacturer's instructions. The protein of interest was visualized using Supersignal West Dura Duration substrate reagent (Thermo, 34080).

### MTT assay

MTT assays were performed in a 96-well plate according to the manufacturer's instructions (Sigma). After the indicated treatments, cells were incubated with MTT at a final concentration of 5 mg/L. After 1–2 hrs, the medium was removed, and the cells were dissolved in MTT solubilisation solution (Sigma). Absorbance at 590 nm (A590) was determined for each well using a microplate reader (Bio-Rad). After subtracting the background absorbance, the A590 of the treated cells was divided by that of the untreated cells to determine the percentage of viable cells.

### Acridine orange staining

In acridine orange-stained cells, the cytoplasm and nucleus appear bright green and dim red, respectively, and acidic compartments appear bright red. The intensity of the red fluorescence is proportional to the degree of acidity. After receiving the specified treatments, cells were incubated with acridine orange solution (1 mg/ml) for 15 min in drug-free medium at 37°C and washed with PBS. Then, cells were trypsinised and analysed by flow cytometry using a FACScan cytometer and CellQuest software, as previously described [20]. Statistical analyses were performed as described above.

### Monodansylcadaverine (MDC) staining

Monodansylcadaverine (MDC) staining was used to quantify the induction of autophagy in microgliatreated with anaerobic. Following treatment, cells were stained with MDC at a final concentration of 10 mM for 10 min at 37°C, collected and fixed in 3% paraformaldehyde in phosphate-buffered saline for 30 min. The cells were then trypsinised and analysed by flow cytometry using a FACScan cytometer and CellQuest software. For each condition, the percentage of cells with characteristic punctate MDC staining indicative of autophagy was assessed.

### Cell death assay

Cells were trypsinised with 0.5 ml 0.25% trypsin for 3 min, collected and resuspended in 1 ml PBS. The cells were then incubated with 0.5 ml staining solution (10 µg/ml PI) at 37°C for 30 min in the dark. Cell death was detected by flow cytometry (BD FACScan Flow cytometer).

### ELISA

The production of IL-8 and TNF-α in the culture supernatants was measured by ELISA as specified by the manufacturer (R&D systems). microgliaplated in 24-well plates were cultured with hypoxia for 6 h. The culture supernatants were aspirated and stored at −70°C until assayed by ELISA. The concentration of IL-8 and TNF-α was determined using a standard curve obtained with IL-8 and TNF-α protein.

### Quantitative RT-PCR

Quantitative RT-PCR analyses for the mRNA were performed by using PrimeScript RT-PCR kits (Takara) as described. The mRNA level of β-actin was used as an internal control. The real-time PCR program steps were 95°C for 1 min, 45cycles of 95°C for 5 s, 60°C for 5 s, and 72°C for 20 s. The sequences of primers used were shown as following: TNF-α, 5′-AGGCGCTCCCCAAGAAGACA-3′ (forward), 5′-TCCT TGGC AAA ACTGCACCT-3′ (reverse); HIF-1α, 5′-TGCTTGGTG CTG AT TTG TG A-3′ (forward), 5′-GGTCAGATGATCAGAGTCCA-3′ (reverse); IL-8, 5′-GGCAGCCTTCCTGATTTCTG-3′ (forward), 5′-GGGGTGGAAAGG TTT GGA GT-3′ (reverse);β-actin, 5′-TTCCTT CCTGGGC ATG G A GTCC-3′ (forward), 5′-TGGCGTACAGGTCTTTGCGG-3′ (reverse).

### Statistical analysis

The results are expressed as the mean ± SD of at least 3 separate experiments performed in triplicate. The differences between groups were determined with the Student's t-tests using SPSS 13.0 software. Differences were declared significant at *P*<0.05. Statistically significant differences are indicated by asterisks (*P*<0.05 (*), *P*<0.01 (**)).

## Results

### Hypoxia induced cell death and inflammation in microglia

**Figure 1:**
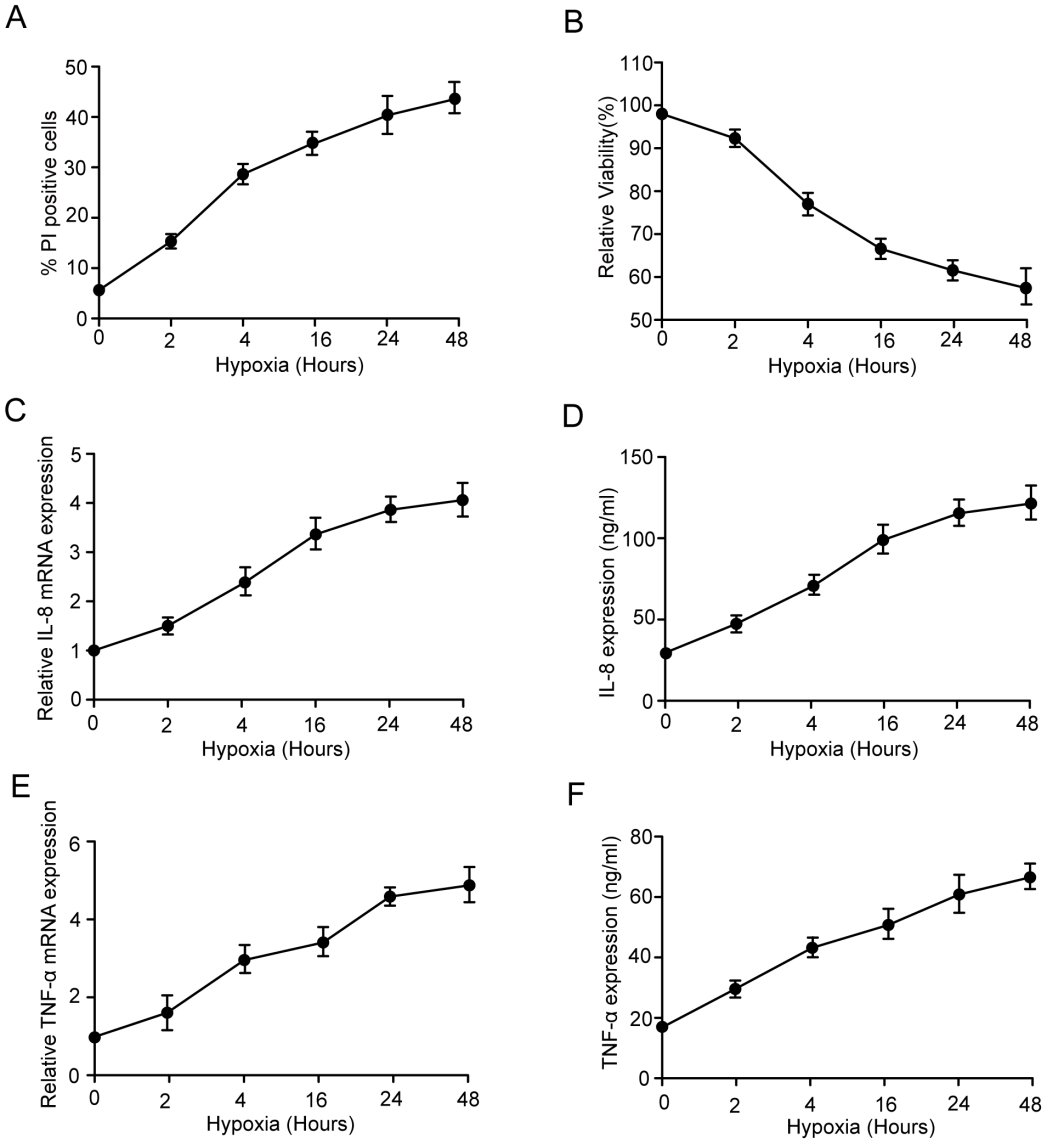
Hypoxia induced cell death and inflammation of microglia cells. (A) To assess cell death in vitro, microglia with the treatment for the indicated time were subjected to PI staining, and analyzed by flow cytometry. The percentage of cells with PI-positive relative to total cell number at each treatment is shown. (B) The effect of hypoxia on the viability of microglia cells. Microglia was treated with the indicated time. Cell viability was assessed using MTT. (C, D, E and F) microglia were treated with the indicated time. The mRNA and protein levels of IL-8 and TNF-α were determined. Experiments performed in triplicate showed consistent results. Data are presented as the mean ±SD of three independent experiments. * *P*<0.05.

To investigate the cell death induced by hypoxia in microglia cells, we first performed a cell death assay, which is considered an effective method for detecting cell death. As shown in Fig. 1A, there were time-dependent decreases in relative cell viability after hypoxia treatment. Similar dose-dependent results were observed in MTT assays (Fig. 1B). These data indicate that hypoxia induces cell death in microglia. Previous studies had shown that hypoxia rapidly induced cell death along with increasing inflammation. Based on the reports, we chose IL-8 and TNF-α as the typical proinflammatory cytokines released by microglia cells in response to hypoxia. As shown in Fig. 1C–F, hypoxia significantly increased the mRNA and protein levels of IL-8 and TNF-α. Taken together, these data indicate that hypoxia induced cell death and inflammation in microglia.

### HIF-1α mediates hypoxia-induced cell death

**Figure 2:**
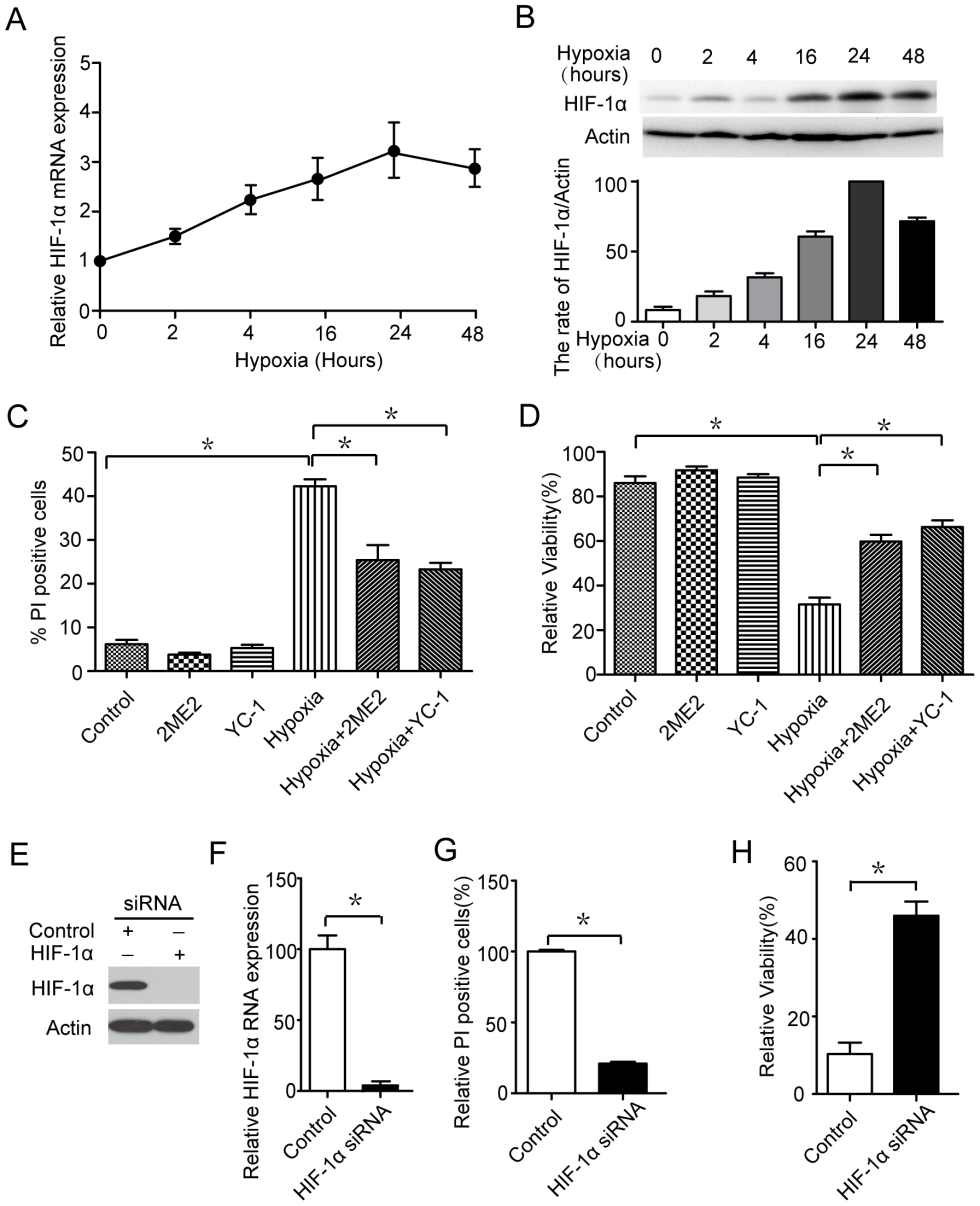
HIF-1 mediates hypoxia-induced cell death. (A) Microglia cells were treated with hypoxia for 0, 2, 4, 16, 24 and 48 h. The kinetics of HIF-1α induction was assayed by qRT-PCR. (B) Measurement of HIF-1α in microglia with the indicated treatments by western blot assay. (C and D) microglia were treated with 3 µM 2ME2, 1 µM YC-1, or the indicated combinations for 6 hours, and the cells were cultured with hypoxia for 16 hrs. The percentage of dead cells was determined using the MTT assay or cell death assay. (E and F) Detection of the inhibition efficiency of siRNAs against HIF-1α. Microglia cells were transfected with siRNAs targeting HIF-1α (100 nM each) for 24 h, and the protein and RNA levels of the target was evaluated by western blot and q-PCR assays. (G and H) The effect of cell death on hypoxia transfected with siRNA control and HIF-1α in microglia (24 h). The % PI positive cells have been normalized to 100% in the control. Experiments performed in triplicate showed consistent results. Data are presented as the mean ± SD of three independent experiments. * *P*<0.05.

HIF-1α, which is a major transcription factor that responds to cellular oxygen reduction, is low under physiological conditions but increase dramatically under hypoxia. We therefore tested whether hypoxia leads to cell death via HIF-1α. As shown in Fig. 2A, there were time-dependent increases of HIF-1α expression after hypoxia treatment. Similar time-dependent results were observed in western blot assay (Fig. 2B). These data indicate that hypoxia induces expression of HIF-1α in microglia. However, we still did not know whether HIF-1α regulated cell death under hypoxia stress. To address it, we exposed the microglia to HIF-1α inhibitors 2ME2 or YC-1,[21], [22] and evaluated the subsequent cell death induced by hypoxia treatment. A significant decrease in hypoxia-induced cell death was observed in microglia after HIF-1α was inhibited (Fig. 2C and 2D). To further address the possibility that the inhibition of HIF-1α is responsible for the cell death induced by hypoxia, we assessed the effects of HIF-1α silencing by RNA interference (Fig. 2E and 2F). The siRNA-mediated knockdown of HIF-1α decreased hypoxia-induced cell death (Fig. 2G and 2H). These data suggested that HIF-1α had an important role in hypoxia-induced cell death.

### HIF-1α mediates hypoxia-induced inflammation

**Figure 3:**
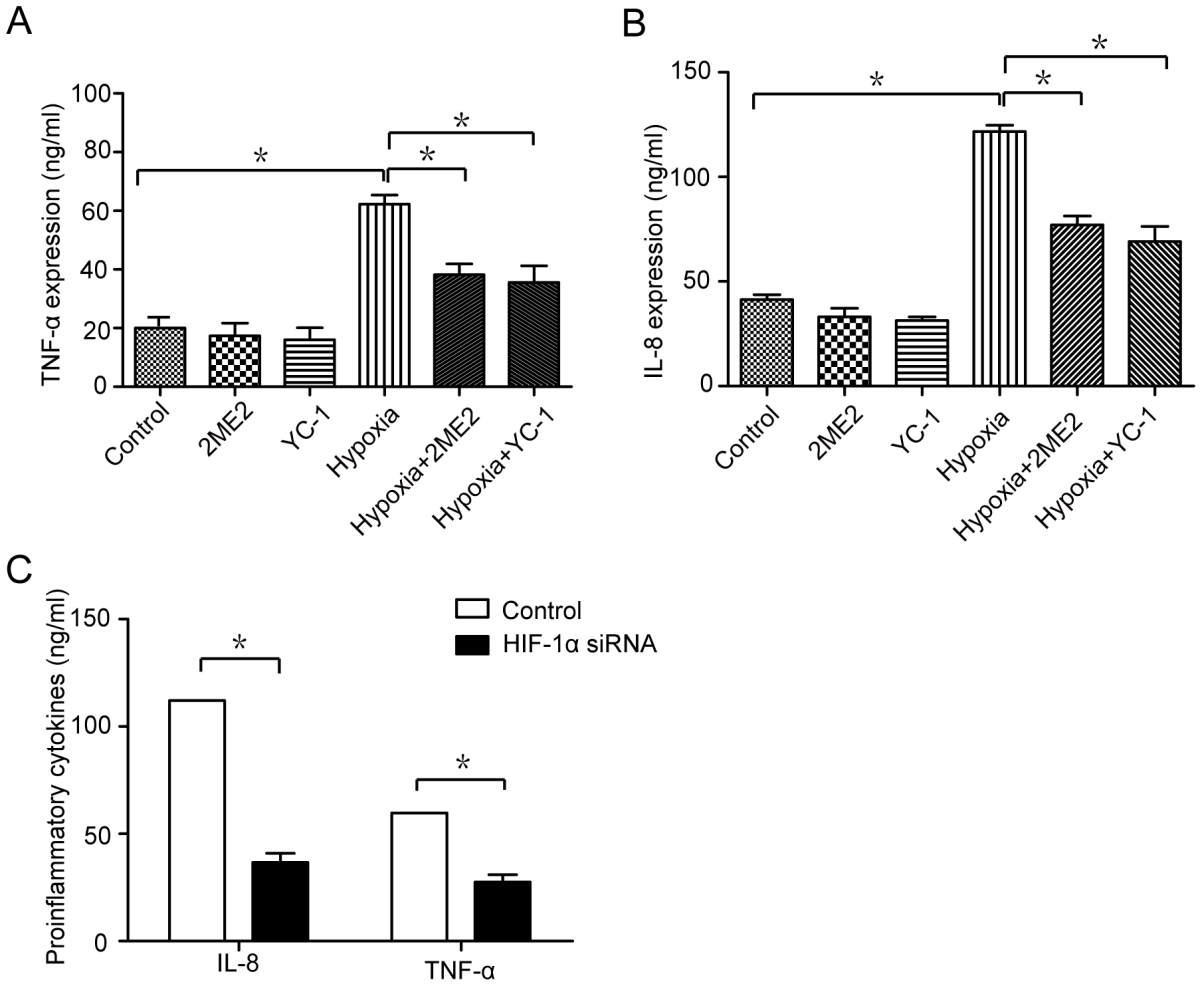
HIF-1 mediates hypoxia-induced inflammation. (A and B) Microglia were treated with 3 µM 2ME2, 1 µM YC-1, or the indicated combinations for 6 hours. The protein levels of IL-8 and TNF-α were determined. (C) Detection of IL-8 and TNF-α by ELISA in microglia transfected with siRNA Con or siRNA HIF-1α (24 h). Experiments performed in triplicate showed consistent results. Data are presented as the mean±SD of three independent experiments. * *P*<0.05.

Given that inflammation is a critical pathogenic factor in response to hypoxic stresses, we asked whether HIF-1α could be responsible for up-regulation of inflammation under hypoxic conditions. We exposed the cells to HIF-1α inhibitors 2ME2 or YC-1, and evaluated proinflammatory cytokines IL-8 and TNF-α induced by hypoxia treatment. A significant decrease in the protein levels of IL-8 and TNF-α was observed in microglia after HIF-1α was inhibited (Fig. 3A and 3B). Furthermore, the siRNA-mediated knockdown of HIF-1α decreased hypoxia-induced proinflammatory cytokines (Fig. 3C). These data suggested that HIF-1α had an important role in hypoxia-induced inflammation.

### Autophagy is induced in microglia after hypoxia treatment

**Figure 4:**
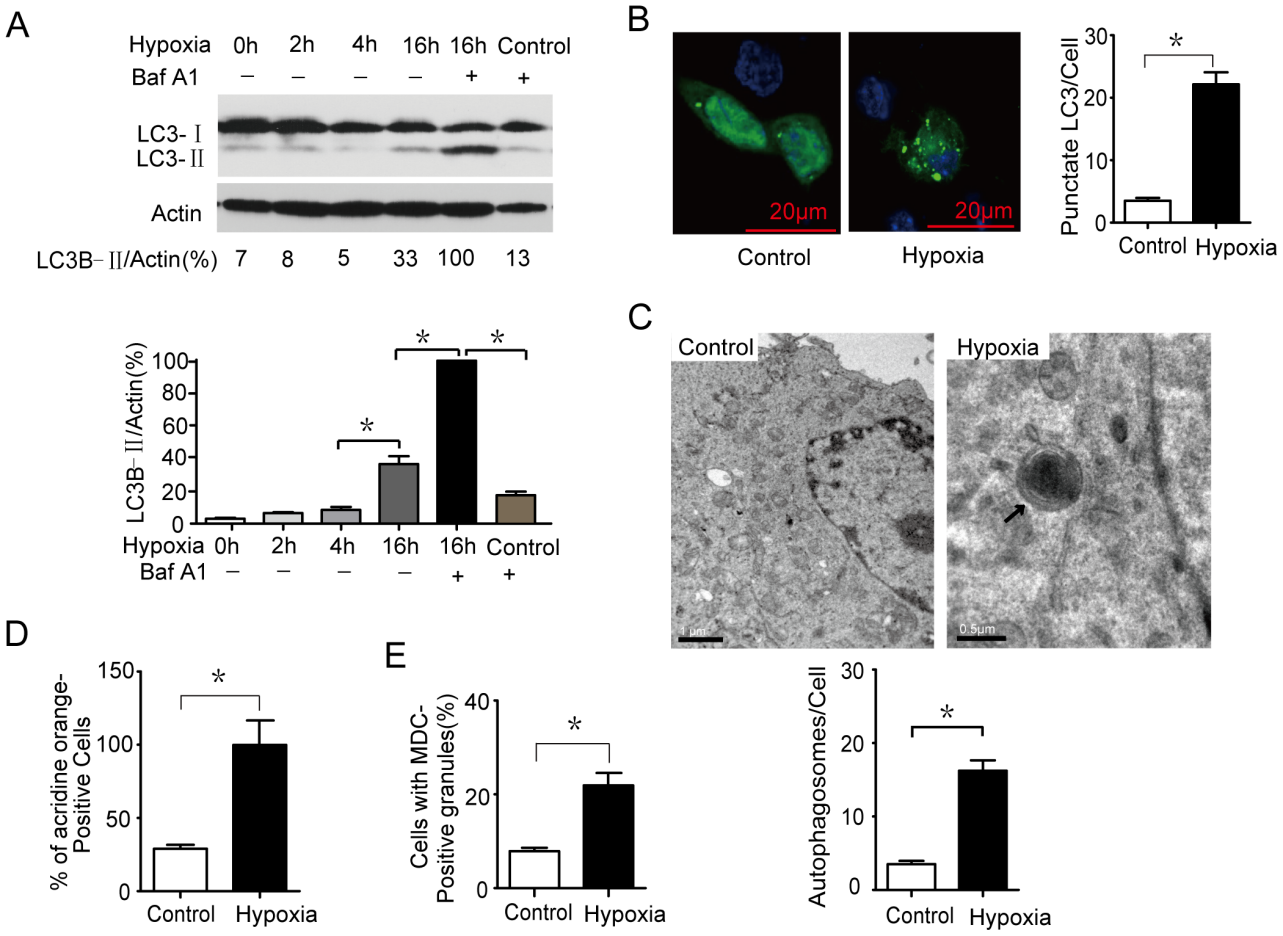
Autophagy is induced in microglia after hypoxia treatment. (A) Hypoxia induced complete autophagic flux in microglia. The cells were treated with hypoxia for the indicated time in the presence of Baf A1 (10 nM). Control represents the normal oxygen conditions. (B) Microglia cells were transfected with a plasmid expressing GFP-MAP1LC3B. After 24 hrs, the cells were exposed to hypoxia for 6 hrs. Cells were visualised by confocal microscopy immediately after fixation. The number of GFP-MAP1LC3B puncta in each cell was counted. (C) Ultrastructural changes in hypoxia-treated microglia. The control shows samples without hypoxia treatment. Closed arrows indicate autophagosomes. (D and E) Microglia were treated with hypoxia for 6 hrs and stained with 1 mg/ml acridine orange or 50 mM MDC for 15 min. After incubation, cells were immediately analysed by flow cytometry. The bar chart demonstrates an increase in mean fluorescent intensity. The asterisks denote significant differences from controls (* *P*<0.05). Experiments performed in triplicate showed consistent results.

Accumulating reports suggested that hypoxia could induce autophagy via HIF-1α. To determine whether autophagy could be induced after hypoxia treatment, we investigated the expression of MAP1LC3B (using Actin as a loading control), which is considered an accurate indicator of autophagy. We observed a gradual increase in the ratio of MAP1LC3B-II to Actin over time in cells treated with hypoxia compared to control cells (Fig. 4A). Furthermore, Baf A1 challenge resulted in the further accumulation of MAP1LC3B-II in microglia after 16 hrs (Fig. 4A), suggesting that hypoxia promotes cellular autophagic flux. To further confirm that hypoxia induces autophagy in microglia, we used a GFP-MAPLC3B puncta formation assay to monitor autophagy. As shown in Fig. 4B, hypoxia-treated microglia displayed a significant increase in the percentage of cells with autophagosomes (GFP- MAPLC3B puncta) after 6 hrs compared with control cells (P<0.05). TEM of microglia treated with hypoxia revealed an increase in the number of autophagosomes (Fig. 4C). Similar results were obtained in acridine orange staining and MDC staining assays (Fig. 4D and 4E). The results illustrate that hypoxia induces a complete autophagic response in microglia.

### Autophagy is involved in hypoxia-induced cell death in microglia

**Figure 5:**
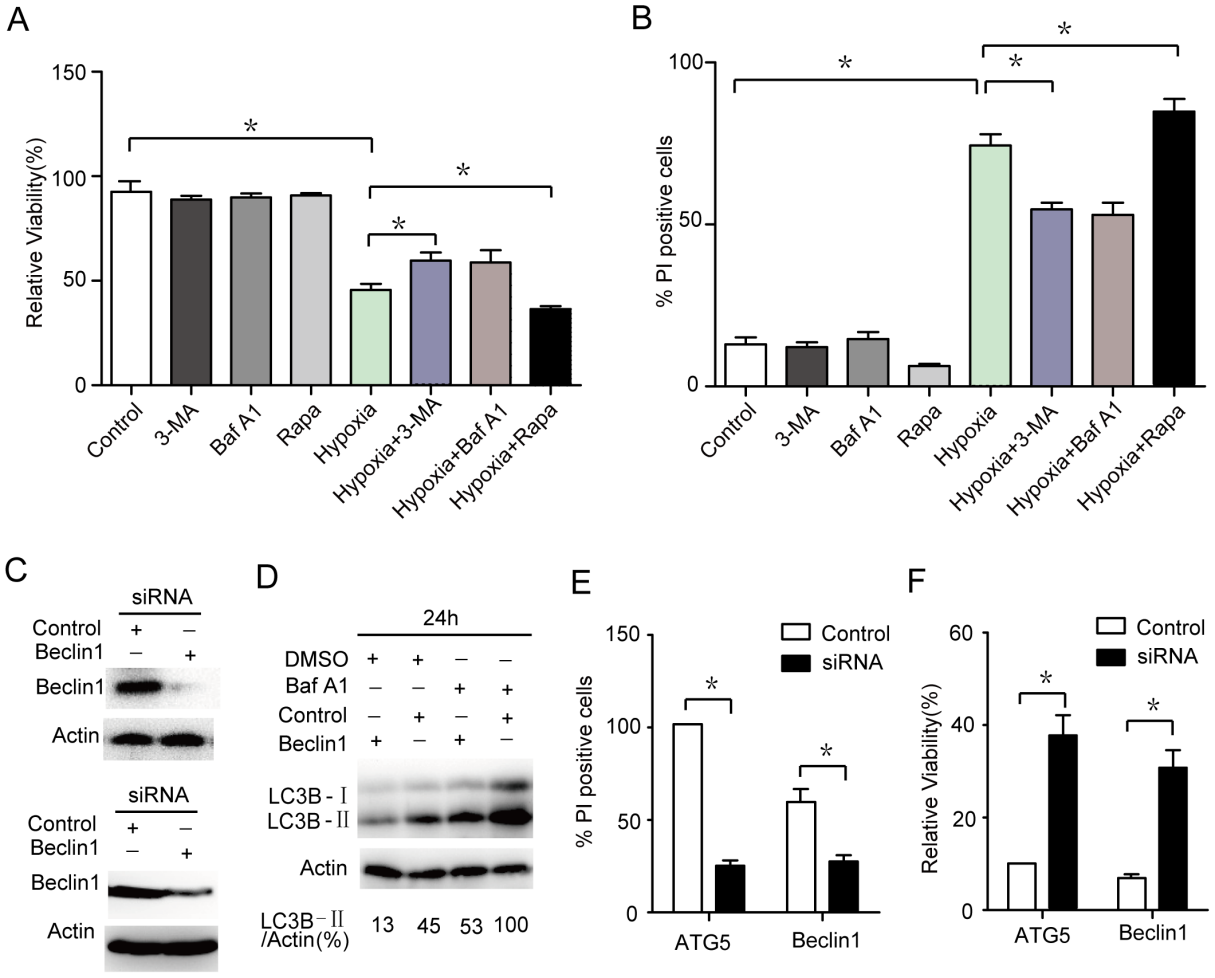
Autophagy is involved in hypoxia-induced cell death in microglia. (A and B) Microglia cells were treated with 100 nM rapamycin, 5 mM 3-MA, 10 nM Baf A1, or the indicated combinations for 24 hrs. After the treatment, the cells were cultured with hypoxia for 6 hrs. The percentage of dead cells was determined using the MTT assay or cell death assay. (C) Detection of the inhibition efficiency of siRNAs against Beclin1 and ATG5. Microglia was transfected with siRNAs targeting Beclin1 and ATG5 (100 nM each) for 24 hrs, and the protein levels of the target were evaluated by western blot. (D) Detection of the inhibition efficiency of LC3 flux with Beclin1 siRNAs. Microglia was transfected with siRNAs targeting Beclin1 and ATG5 (100 nM each), and treated with Baf A1 for 24 hrs. The protein levels of the target were evaluated by western blot. (E and F) MTT and cell death assays measuring the hypoxia-treated cell death ratio after transfection with siRNAs against Beclin1 and ATG5. Experiments performed in triplicate showed consistent results. Data are presented as the mean ±SD of three independent experiments. * *P*<0.05.

Autophagy is enhanced under stress conditions, and it can promote cell survival or cell death depending on the type of cellular stress. To determine whether hypoxia-induced autophagy promotes microglia survival or death, we exposed the cells to autophagy inhibitors (3-methyladenine, 3-MA; bafilomycin A1, Baf A1) or activators (rapamycin, Rapa), and evaluated the subsequent cell death induced by hypoxia treatment. A significant decrease in hypoxia-induced cell death was observed in microglia after autophagy was inhibited (3-MA or Baf A1 treatment), while a decrease was observed in microglia when autophagy was induced (Rapa treatment) (Fig. 5A and 5B). To further address the possibility that the inhibition of autophagy is responsible for the cell death induced by hypoxia, we assessed the effects of Beclin1 and ATG5 silencing and autophagy flux by RNA interference (Fig. 5C and 5D). The siRNA-mediated knockdown of Beclin1 and ATG5, which are required for autophagy, decreased hypoxia-induced cell death (Fig. 5E and 5F), suggesting that autophagy promotes the cell death induced by hypoxia.

### HIF-1α mediates microglia autophagy induced by hypoxia

**Figure 6:**
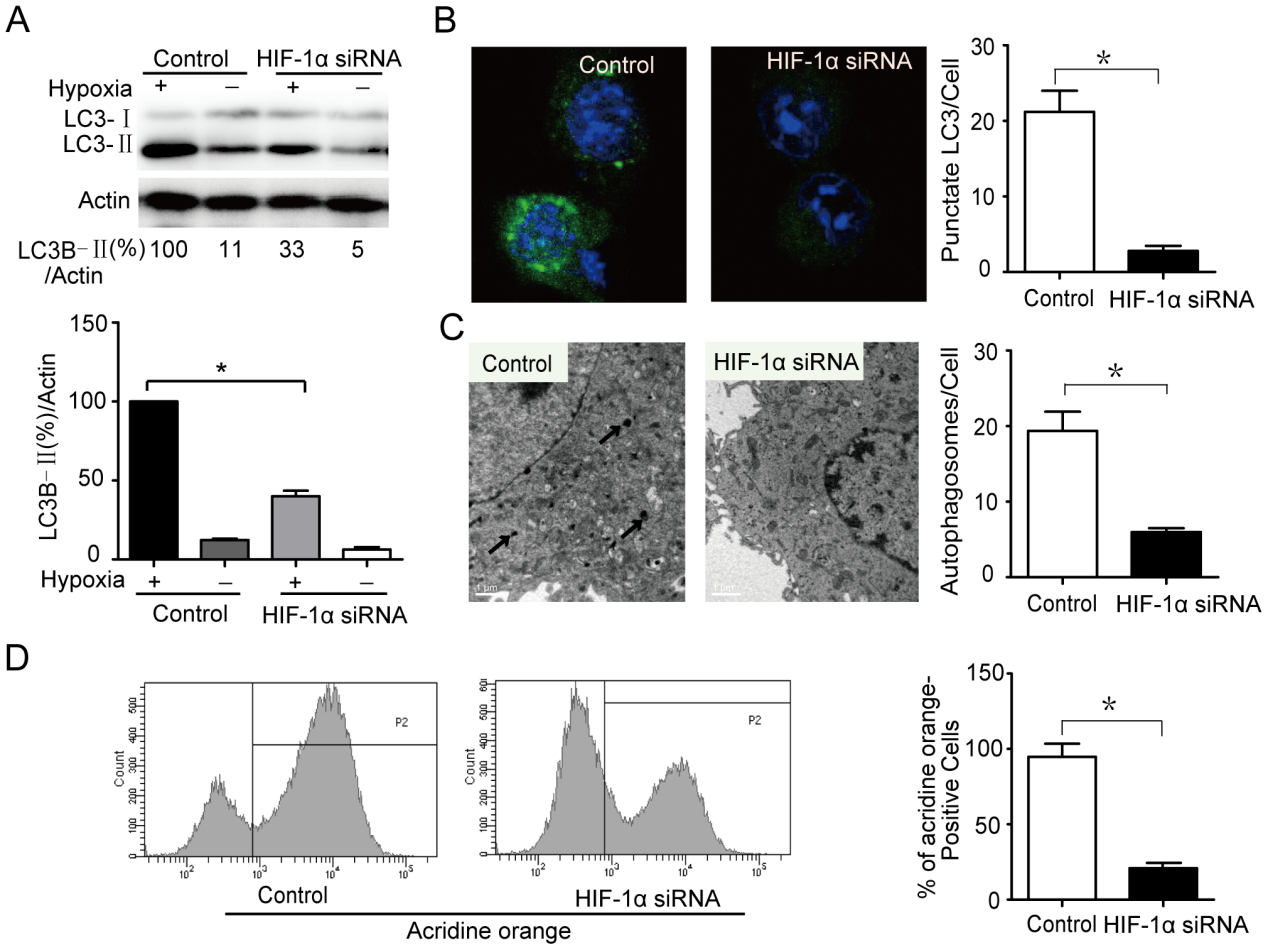
HIF-1α mediates microglia autophagy induced by hypoxia. (A) Microglia cells were transfected with siRNAs targeting HIF-1α (100 nM each) for 24 hrs following hypoxia treatment for 16 hrs, and then the protein levels of the target were evaluated by western blot. (B and C) Confocal microscopy and electron microscopy detected autophagosomes. (D) Microglia cells were transfected with siRNA HIF-1α, after treated with hypoxia for 16 hrs. Flow cytometry detected acridine orange positive cell. The asterisks denote significant differences from controls (* *P*<0.05). Experiments performed in triplicate showed consistent results.

Although accumulating studies have reported that HIF-1α could mediate autophagy in different diseases, it is not clear whether HIF-1α mediates microglia autophagy induced by hypoxia. As shown in Fig. 6A, there was a significant decrease in the ratio of LC3B-□ to Actin in transfected HIF-1α siRNA cells following hypoxia treatment. The formation of GFP-LC3 puncta is another marker of autophagosomes. As shown in Fig. 6B, HIF-1α siRNA decreased the number of GFP-PLC3 puncta in microglia after hypoxia treatment (P<0.05). TEM analysis also revealed a decrease in the number of autophagosomes in the microglia transfected with HIF-1α siRNA (Fig. 6C). Consistent with these results, acridine orange staining assays had significant changes (Fig. 6D). These indicate that HIF-1α mediates microglia autophagy under hypoxia treatment.

## Discussion

In current study, we firstly found that hypoxia induces autophagic cell death by upregulation of HIF-1α in microglia cells. The finding is supported by the following evidence: (1) hypoxia induced microglia death and inflammation; (2) HIF-1α mediates hypoxia-induced cell death and inflammation; (3) autophagy is involved in hypoxia-induced cell death in microglia; and (4) HIF-1α mediates microglia autophagy induced by hypoxia.

Stroke is one of the most frequent causes of death and disability worldwide. Cerebral ischemia is the major insult of stroke and induces microglia mediated acute inflammation by triggering excessive production of proinflammatory cytokines, leading to the exacerbation of primary brain damage [23], [24], [25]. However, the pathways of microglia in controlling proinflammatory cytokines have not been well addressed during cerebral ischemia.

In our study, to investigate the cell death induced by hypoxia in microglia cells, we first performed a cell death assay (Fig. 1A and B), and detected the proinflammatory cytokines (Fig. 1C–F). These data indicate that, consistent with other reports, hypoxia rapidly induced cell death along with increasing inflammation.

As for the molecular mechanism, transcriptional factor hypoxia- inducible factor 1 (HIF-1) is considered to play fundamental role under hypoxia [26], [27], [28]. At early stage of hypoxia, induction of HIF-1 could serve to protect cells from destruction [29], [30]. We tested whether hypoxia leads to cell death via HIF-1α. As shown in Fig. 2A and B, there were time-dependent increases of HIF-1α expression after hypoxia treatment. In addition, we exposed the microglia to HIF-1α inhibitors and HIF-1α siRNA (Fig. 2 C–E and Fig. 3), to evaluate the subsequent cell death and proinflammatory cytokines induced by hypoxia treatment. The results demonstrated that HIF-1α inhibition could significantly decrease in hypoxia-induced cell death and proinflammatory cytokines. Therefore, HIF-1α is responsible for the cell death and inflammation induced by hypoxia.

Autophagy is induced under conditions of stress such as starvation, hypoxia, heat, and drug treatment [31]. Autophagy has been associated with both cell survival and cell death, but the role of autophagy in cell death has been controversial [31]. HIF-1, by regulating the expression of its target downstream BNIP3 and BNIP3L, regulates the autophagy under hypoxia [32], [33], [34]. Recent studies indicate that BNIP3 induces autophagy by disrupting the interaction of Beclin 1 with Bcl-2 and Bcl-XL [35]. On the contrary, it is reported that oxygen deprivation-induced autophagy did not require HIF-1 activity [36]. Therefore, to further identify whether autophagy could be induced after hypoxia treatment, we investigated the autophagy formation and the specific role in microglia survival or death. The results illustrate that hypoxia induces a complete autophagic response in microglia (Fig. 4). In addition, we also exposed microglia to autophagy inhibitors, activators or Beclin1 RNAi, and evaluated the subsequent cell death induced by hypoxia treatment. We found that autophagy promotes the cell death via Beclin1 induced by hypoxia (Fig. 5). Through transfected with siRNAs targeting HIF-1α, it is found that HIF-1α mediates microglia autophagy under hypoxia treatment (Fig. 6).

In summary, our findings provide a novel mechanism that hypoxia induces autophagic cell death by upregulation of hypoxia-inducible factor HIF-1α in microglia, and HIF-1α affects autophagy-related events might through activation of Beclin1. In addition, these results may represnt a potential therapeutic avenue for the treatment of ischemic/hypoxic brain injury.
